# Comparative Genomics of the Baltic Sea Toxic Cyanobacteria *Nodularia spumigena* UHCC 0039 and Its Response to Varying Salinity

**DOI:** 10.3389/fmicb.2018.00356

**Published:** 2018-03-08

**Authors:** Jonna E. Teikari, Shengwei Hou, Matti Wahlsten, Wolfgang R. Hess, Kaarina Sivonen

**Affiliations:** ^1^Department of Microbiology, University of Helsinki, Helsinki, Finland; ^2^Genetics and Experimental Bioinformatics, Institute of Biology III, University of Freiburg, Freiburg, Germany; ^3^Freiburg Institute for Advanced Studies, University of Freiburg, Freiburg, Germany

**Keywords:** Baltic Sea, cyanobacteria, *Nodularia spumigena*, comparative genomics, RNA sequencing, salinity

## Abstract

Salinity is an important abiotic factor controlling the distribution and abundance of *Nodularia spumigena*, the dominating diazotrophic and toxic phototroph, in the brackish water cyanobacterial blooms of the Baltic Sea. To expand the available genomic information for brackish water cyanobacteria, we sequenced the isolate *Nodularia spumigena* UHCC 0039 using an Illumina-SMRT hybrid sequencing approach, revealing a chromosome of 5,294,286 base pairs (bp) and a single plasmid of 92,326 bp. Comparative genomics in *Nostocales* showed pronounced genetic similarity among *Nodularia spumigena* strains evidencing their short evolutionary history. The studied Baltic Sea strains share similar sets of CRISPR-Cas cassettes and a higher number of insertion sequence (IS) elements compared to *Nodularia spumigena* CENA596 isolated from a shrimp production pond in Brazil. *Nodularia spumigena* UHCC 0039 proliferated similarly at three tested salinities, whereas the lack of salt inhibited its growth and triggered transcriptome remodeling, including the up-regulation of five sigma factors and the down-regulation of two other sigma factors, one of which is specific for strain UHCC 0039. Down-regulated genes additionally included a large genetic region for the synthesis of two yet unidentified natural products. Our results indicate a remarkable plasticity of the *Nodularia* salinity acclimation, and thus salinity strongly impacts the intensity and distribution of cyanobacterial blooms in the Baltic Sea.

## Introduction

Photoautotrophic cyanobacteria have adapted to life in a wide range of aquatic and terrestrial habitats. In aquatic environments, salinity (referring here to the concentration of dissolved NaCl) is an important abiotic factor influencing prevailing cyanobacterial species distribution (Sivonen et al., 2007; Pade and Hagemann, 2014; Celepli et al., 2017). Proper strain-specific concentrations of inorganic salts have a crucial role in maintaining constant turgor pressure, while fluctuating salinity changes the water potential and inorganic ion concentration in cells (Hagemann, 2011). A rapid salinity shift triggers complex acclimation processes that keep osmotic equilibrium and inorganic ion concentrations suitable for proper cellular function and growth. Response to salt shock is well-understood in the model cyanobacterium *Synechocystis* sp. strain PCC 6803 (Kanesaki et al., 2002; Marin et al., 2002, 2003, 2004; Huang et al., 2006; Nikkinen et al., 2012; Qiao et al., 2013). Early-stage adaptation (<1 h) is based mainly on the activation/inactivation of existing transporters and the expression of numerous salt stress-related genes, such as genes for the synthesis of compatible solutes to diminish cellular damage, occurs within 24 h (Hagemann and Marin, 1999; Marin et al., 2003; Fulda et al., 2006). Increased salt concentration additionally induces general stress responses such as the synthesis of chaperones and the arrest of photosynthetic activity and cell division (Kanesaki et al., 2002; Fulda et al., 2006; Qiao et al., 2013; Billis et al., 2014; Rai et al., 2014). Much less is known of how lower salinity affects cyanobacterial molecular responses and adaptation.

The semi-enclosed Baltic Sea is one of the largest brackish water ecosystems in the world. The salinity gradient in the Baltic Sea basins is dynamic due to the mixture of inflowing freshwater from the large catchment area and saline water pulses via the Danish Straits. The Baltic Sea is well-known for the toxic cyanobacterial blooms appearing each summer, causing not only health risks for humans and animals, but also substantial economic losses (e.g., recreation and tourism). Increased temperature, nutrient bioavailability, and thermal stratification are generally agreed to promote the formation and intensity of cyanobacterial blooms (Paerl and Huisman, 2008; Conley et al., 2009). Cyanobacterial blooms in the Baltic Sea are dominated by filamentous and nitrogen-fixing (diazotrophic) genera *Nodularia spumigena, Aphanizomenon* and, to a lesser extent, *Dolichospermum* sp. (previously *Anabaena*) (Halinen et al., 2007; Sivonen et al., 2007; Fewer et al., 2009), of which *Nodularia spumigena* and *Dolichospermum* may produce toxins, such as nodularin and microcystins (Sivonen et al., 1989; Halinen et al., 2007). Climate-change models indicate that salinity in the Baltic Sea will decrease due to rarer saline pulses and elevated riverine runoff (Kjellström and Ruosteenoja, 2007; von Storch et al., 2015; Graham, 2016). Decreased salinity may shift the distribution of freshwater species further south and promote the formation of freshwater origin *Dolichospermum* blooms (Brutemark et al., 2015; Vuorinen et al., 2015). Despite the intensive research on Baltic Sea cyanobacterial blooms, only one draft genome of a Baltic Sea cyanobacterial isolate has been sequenced and the availability of genomic information for brackish water cyanobacteria has remained scarce (Voß et al., 2013; Celepli et al., 2017). The previously studied *Nodularia spumigena* CCY9414 was isolated from the surface water near Bornholm, in the southern Baltic. In our study we sequenced the genome of *Nodularia spumigena* strain UHCC 0039, isolated from the open Gulf of Finland (Sivonen et al., 1989) in the northern Baltic, to gain better genomic coverage of Baltic Sea phototrophs and to increase understanding of the cyanobacterial acclimation strategies in a dynamic brackish water environment (salinity range 0–9 mg/L). An RNA-Seq based transcriptomic study was conducted to unravel transcriptional responses of *Nodularia spumigena* to salinity changes relevant in the Gulf of Finland.

## Materials and methods

### Strains and toxin analysis

The nodularin-producing *Nodularia spumigena* strain UHCC 0039 (hereafter *Nodularia* UHCC 0039) belongs to the University of Helsinki culture collection (HAMBI, UHCC). *Nodularia* UHCC 0039 (former name *Nodularia spumigena* AV1) was isolated from the open Gulf of Finland (Sivonen et al., 1989). The strain was purified to axenicity, and has been cultivated in Z8 medium without nitrogen (Kotai, 1972) under continuous illumination since isolation. Three replicates of *Nodularia* UHCC 0039 were inoculated to separate cell culture flasks (TC Flask T175, Sarsted AG & Co) with 115 mL of Z8X medium containing 0.321; 3.023; 6.047 or 9.071 g L^−1^ NaCl. Salinities corresponded to conductivities of 0.83; 5.55; 10.10; 14.37 mS/cm, and will further be referred as 0, 3, 6 and 9 g L^−1^ NaCl, respectively. Cultures were grown at 20 °C under continuous illumination of 3.2–3.7 μmol photons m^−2^s^−1^ for 24 days. To follow the growth of the cultures chlorophyll *a* concentration was determined in every fourth day during the experiment. 1 mL of cell culture was collected on glass microfiber filters (GF/F, Glass Microfiber Binder Free, GE Healthcare) and stored at −80 °C. Chlorophyll *a* was extracted for 24 h at −20 °C with acetone and absorptions of the extracts were measured at 664, 630, and 647 nm. Chlorophyll *a* concentration was calculated using the equation of Jeffrey and Humphrey (Jeffrey and Humphrey, 1975). In addition, number of cells was estimated from the cultures containing 0 and 6 g L^−1^ NaCl every fourth day. Cells were fixed in 5% of Lugol's solution and stored at +4 °C. Total length of cellular filaments in 0.5 μl droplet was measured and divided by average cell size.

#### Toxin analysis

Combined intra- and extracellular concentrations of nodularin were determined by extracting toxins from freeze-dried samples to 70% methanol at 80 °C for 1 hour. Samples were injected into Acquity ultra performance liquid chromatography (UPLC) system (Waters, Manchester, UK), equipped with Kinetex® 1.7 μm C8 100 Å, 50 × 2.1 mm LC Column. The UPLC was operated with a flow-rate of 0.3 ml/min in gradient mode, at a temperature of 40 °C. Solvents used in the gradient were A: 0.1% formic acid in water and B: 0.1% formic acid in 1 to 1 mixture of acetonitrile and isopropanol. The initial conditions of the linear gradient were A: 25% and B: 75% and the conditions were changed to A: 35% and B: 65% in 5 min. Injection volume was 1 μL. Mass spectra were recorded with Waters SynaptG2-Si mass spectrometer (Waters, Manchester, UK). Measurements were performed using negative electrospray ionization (ESI) in resolution mode and ions were scanned in the range from 500 to 1,300 m/z. MS analyses were performed with scan time of 0.1 s. Capillary voltage was 2.0 kV, source temperature 120 °C, sampling cone 40.0, source offset 80.0, desolvation temperature 600 °C, desolvation gas flow 1,000 L/h and nebulizer gas flow 6.5 Bar. Leucine-encephalin was used as a lock mass and calibration was done with mixture of sodium formate and ultramark® 1621. Standard curve containing dilution series of known nodularin concentrations was run alongside toxin samples. All the samples, including standards, were spiked by nostophycin to ensure successful extraction and device functioning.

### Comparative genomics

Genomic DNA of *Nodularia* UHCC 0039 was extracted using a NucleoBond® AXG kit (Macherey Nagel) following the manufacturer's instructions. DNA libraries, PacBio RS II sequencing with P6-C4 chemistry and genome assembly using HGAP3 protocol with Quiver polishing (Chin et al., 2013) were conducted in the DNA Sequencing and Genomics Laboratory, Institute of Biotechnology, University of Helsinki. To reinforce genome assembly, paired-end Illumina HiSeq2500 reads (Macrogen, Inc.) were used to correct PacBio assemblies using Pilon v1.20 software (Walker et al., 2014). Genomes were annotated using Prokka v1.12 (Seemann, 2014). A total of 28 complete and draft cyanobacteria genomes were included in the comparative genomics study. Detailed information of the bioinformatics workflow is provided in the Supporting information.

### Transcriptome sequencing and analysis

Total RNA from the cultures containing 0 or 6 g L^−1^ NaCl was isolated on day 16 using the RNeasy mini kit (Qiagen) and genomic DNA was degraded using the TURBO DNA-*free*™ kit (Life Technologies). Paired-end cDNA libraries were analyzed with an Illumina HiSeq sequencer at the Institute for Molecular Medicine Finland (FIMM). RNA-Seq data analysis was mainly performed on a 64-cores local Unix server and computation-intensive read alignment steps were performed on the Galaxy instance of Freiburg University (Cock et al., 2013; Afgan et al., 2016). In short, the quality of the raw reads was checked using FastQC v0.11.4 (Andrews, 2010), and reads were demultiplexed with Trimmomatic (Bolger et al., 2014). Clean reads were aligned to reference genomes using BWA-MEM v0.7.7 (Li, 2013) in paired-end mode with default parameters. The read count per gene were calculated using featureCounts v1.4.6.p5 (Liao et al., 2014). The differentially expressed genes (false discovery rate (FDR) < 0.01) between control (6 g L^−1^ NaCl) and treatment (0 g L^−1^ NaCl), were called using both edgeR (Robinson et al., 2010) and DESeq (Anders and Huber, 2010) following the simple design protocol (Anders et al., 2013). Detailed descriptions of the workflow and parameters are provided in the Supporting information.

### Data deposition

Raw reads, genome assemblies, and annotations were deposited in NCBI under the BioProject accession number of PRJNA352241.

## Results

### Genomic characteristics of *Nodularia spumigena* UHCC 0039

The whole-genome assembly of *Nodularia* UHCC 0039 resulted in two circular contigs, corresponding to the chromosome (5.29 Mbp) and one plasmid (0.92 Mbp) (Table S1). The average coverage of PacBio reads was 368 × for the chromosome and 384 × for the plasmid. The PacBio assembly was polished by Illumina sequencing, yielding an average coverage of 252 × for the chromosome and 255 × for the plasmid, which corrected 14 single nucleotide polymorphisms (SNP), 5 insertions, and 241 deletions on the chromosome and 2 insertions and 19 deletions on the plasmid, respectively. The final genome size of *Nodularia* UHCC 0039 is 5.38 Mbp. We modeled 5,108 protein-coding genes, 2,699 of which were annotated encoding hypothetical proteins. This relatively high share of genes lacking functional annotation illustrates the uniqueness of the *Nodularia* group.

**Figure 1:**
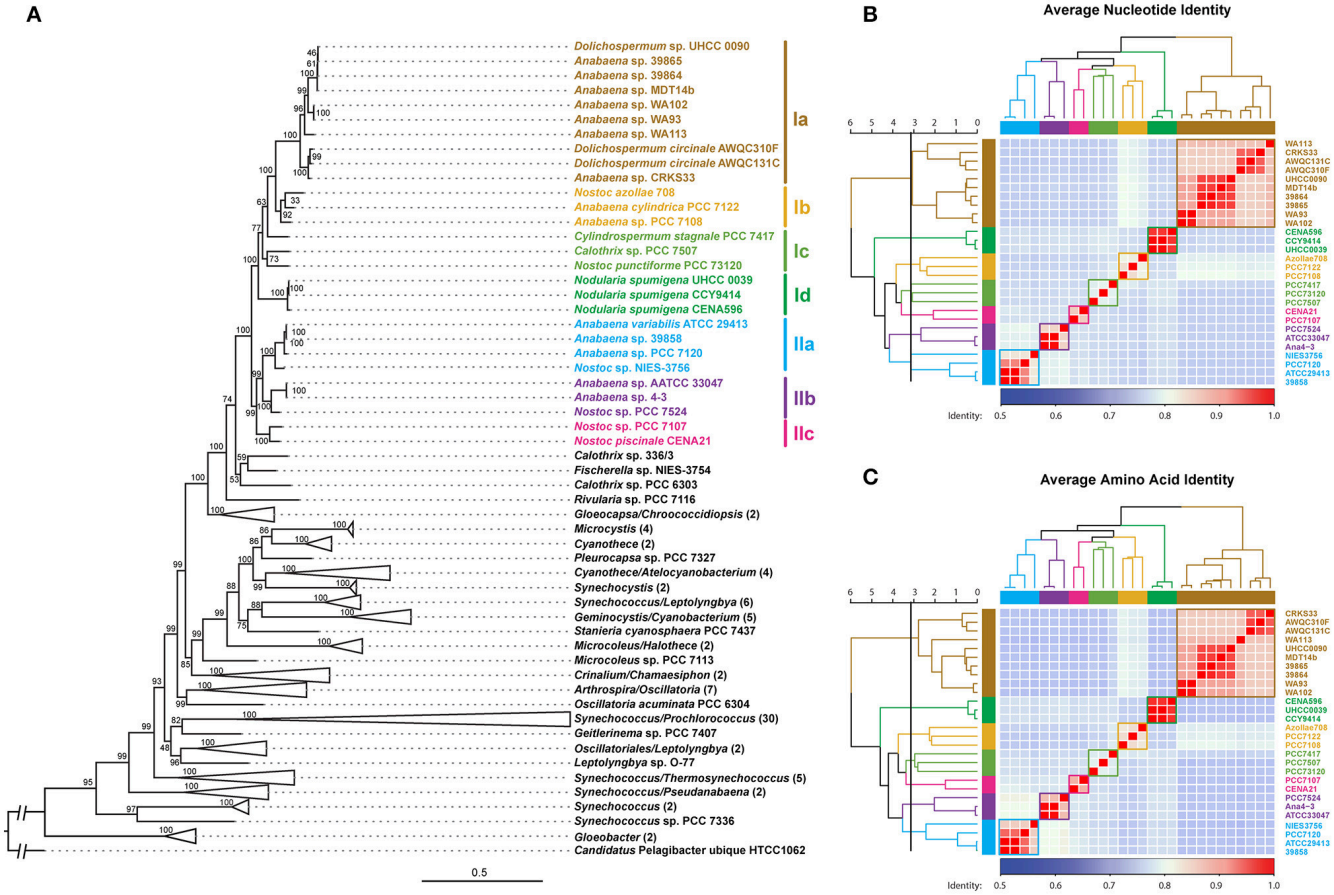
Phylogenomic placement **(A)**, average nucleotide **(B)**, and amino acid **(C)** identities within sequenced *Nostocales*. The maximum-likelihood phylogenomic tree was constructed based on the concatenated alignment of 31 universal marker genes (Wu and Eisen, 2008) and was rooted by using Candidatus *Pelagibacter ubique* HTCC1062 as the outgroup. For simplicity and clarity, branches with a bootstrap value of 100 were collapsed except for *Nostocales*. The Average Nucleotide Identity (ANI) and Average Amino Acid Identity (AAI) were clustered using average linkage hierarchical clustering based on pairwise Euclidean distances. Seven clusters were determined by cutting the dendrograms at a tree height of 3.2, the corresponding phylogenetic relationship and cluster memberships are shown on the phylogenomic tree.

**Figure 2:**
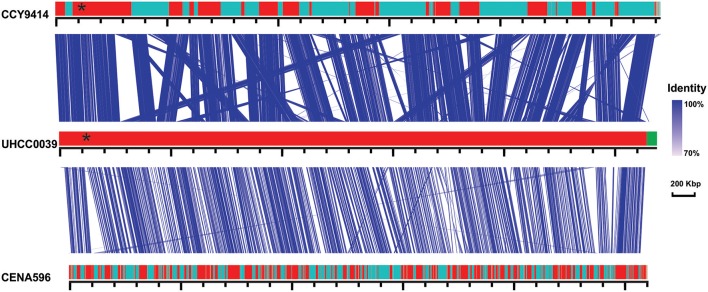
Similarities among genomic contigs of *Nodularia spumigena* strains. Previously defined contigs of CCY9414 and CENA596 were aligned and reordered according to the UHCC 0039 genome assembly. Contigs were colored in alternating red and cyan colors. Similar regions within contigs inferred by BlastN are connected by blue lines. Plasmid pUHCC0039a is indicated by the green box. The asterisks (*) indicate the location of a *phn* gene cluster located at 535,027–547,520 bp for the metabolism of phosphonates (Teikari et al., 2018), that is highly similar to a region in strain CCY9414. The visualization is only valid for the respective contigs and cannot be taken for total genome comparison because only the UHCC 0039 genome sequence is finished whereas the other genome assemblies consist of multiple contigs (cf. Table S1).

A phylogenomic tree was constructed for 120 selected cyanobacteria genomes based on 31 conserved marker genes (Figure 1A; Wu and Eisen, 2008). We additionally calculated the Average Nucleotide Identity (ANI) (Table S2) and Average Amino Acid Identity (AAI) (Table S3). Twenty-eight *Nostocales* genomes formed seven different clusters (Figures 1B,C), partitioning the *Nostocales* branch into seven corresponding subgroups (Ia–d; IIa–c) in the phylogenomic tree (Figure 1A). All *Nodularia spumigena* strains clustered tightly together within subgroup Id, indicating the congruent evolutionary history of these brackish-water specialists. The largest subgroup Ia consisted of *Dolichospermum*/*Anabaena* species, for which larger within-group variation was observed, indicating their faster divergent evolution. Despite the high within-group similarity of the three *Nodularia* strains, specific differences were observed when collinear blocks were compared for local synteny (Figure 2). Synteny at contig level was more pronounced between *Nodularia spumigena* UHCC 0039 and its Baltic Sea counterpart *Nodularia spumigena* CCY9414, including a region for the metabolism of phosphonates (Teikari et al., 2018). Moreover, sequences homologous to plasmid pUHCC0039a were detected in strain CCY9414 but not CENA596 (Figure 2). This is consistent with their close geographical and evolutionary relationship, compared to *Nodularia spumigena* CENA596, which was isolated from a shrimp production pond in Brazil (Popin et al., 2016). However, synteny cannot be considered at total genome level because only the UHCC 0039 genome sequence was finished.

**Figure 3:**
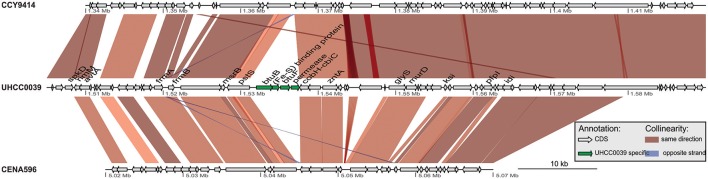
Gene cassette encoding vitamin B12 receptor and ligand-gated transport channel.

The detailed comparison of *Nodularia spumigena* genomes revealed several strain-specific insertions into the UHCC 0039 genome among otherwise syntenic regions that are likely of ecological relevance. The four-gene cassette BMF81_01278 to BMF81_01281 contains the genes *btuB* and *btuF*, which constitute an outer membrane vitamin B12 receptor and ligand-gated transport channel (Figure 3). This cassette is immediately upstream of the conserved *cobH-cbiC* gene BMF81_01282 encoding precorrin-8X methylmutase, an enzyme in the (pseudo)cobalamin (vitamin B12) synthesis pathway. Numerous marine microbes depend on certain other microbes to meet their vitamin B12 demand, consistent with the presence of secretion and uptake systems in many of these species (Bonnet et al., 2010; Heal et al., 2017). Marine cyanobacteria appear to synthesize pseudocobalamin, a form not commonly bioactive in eukaryotic algae, but certain species can remodel it to the active cobalamin form (Helliwell et al., 2016). Therefore, the addition of an import system for vitamin B12 in strain UHCC 0039 adds complexity to this topic, as it can release the requirement to synthesize B12 vitamin for itself. Other interesting gene insertions include the BMF81_03816 to BMF81_03843 cassette for surface-modifying enzymes (including several glycosyl transferases, epimerases, and acetylglucosamine-modifying enzymes) and the genes BMF81_01521 to BMF81_01525 encoding a distinct set of chaperones, co-chaperones, and peptidases (Figure S1).

Insertion sequences (IS) are a driving force for genome evolution (Mahillon and Chandler, 1998). A genome-wide search of IS elements in the three *Nodularia* strains unraveled that Baltic Sea *Nodularia spumigena* carry more candidates (181 for CCY9414 and 148 for UHCC 0039) than their Brazilian counterpart CENA596 (71) (Figure S2a). A PCA analysis of the IS elements among three *Nodularia* strains showed that the Baltic Sea strains were well separated from the Brazilian strain on the first component, which explained 80.3% of the variance. The most frequent IS family is IS200/IS605 (64/55/21 for CCY9414, UHCC 0039, and CENA596), followed by IS4 (25/22/6), IS607 (19/20/2), IS5 (13/11/6), and IS630 (24/2/7). The Brazilian *Nodularia spumigena* strain encodes two IS1, three IS982, and one ISH3 that were not found in the Baltic Sea isolate. Together with IS630, IS701 (3/8/3) and IS91 (0/2/0) were the driving families that separated the two Baltic Sea strains on the second principal component (Figure S2b).

### Specific and shared proteins in the genus *Nodularia*

**Figure 4:**
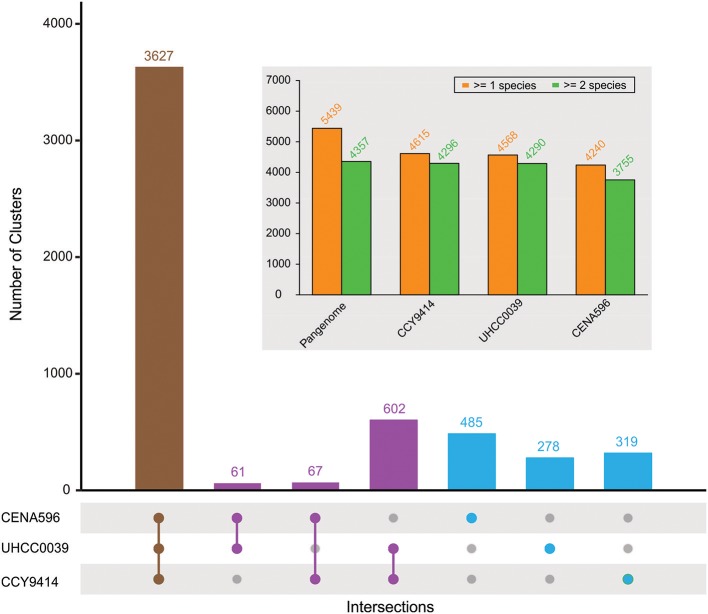
Pan-genome composition of three sequenced *Nodularia* strains. The number of gene clusters is given for each type of intersection. The total number of gene clusters found in at least one or two species for the pan-genome and for each strain are shown in the subplot.

The comparison of the predicted proteomes revealed 3627 gene clusters shared by the three strains (Figure 4). In total, 5439 protein clusters were determined, 4375 clusters of which were shared by at least two species (Table S4). Pangenome analysis revealed that 730 clusters were shared by two *Nodularia* strains, 82.54% of them belonging to *Nodularia* CCY9414 and *Nodularia* UHCC 0039, indicating close gene composition between these two strains. Among the 1,082 orphan clusters, *Nodularia* CENA596 took up of 44.8%, followed by *Nodularia* CCY9414 (29.5%), and only 278 orphan clusters were detected in *Nodularia* UHCC 0039. Previously, 1,098 potentially unique proteins were found in *Nodularia spumigena* CCY9414 that were not present in other *Nostocales* (Voß et al., 2013). Many of these putative proteins are smaller than 80 amino acids, a systematically underestimated class of gene products in bacteria. However, several studies revealed the involvement of small proteins in essential regulatory and other processes (Storz et al., 2014). The detection and functional characterization of small proteins in cyanobacteria has received particular attention (Baumgartner et al., 2016). We therefore decided to add their genes, if they were conserved and had a minimum length of 50 amino acids, to the other two genomes, leading to the numbers presented in Figure 4.

Among the shared and strongly conserved genes, we found a gene cluster putatively involved in the biogenesis of chaperone-usher (CU) fimbriae, the cell surface-located organelles found in many Gram-negative bacteria (Waksman and Hultgren, 2009; Wurpel et al., 2013). These genes encode a fimbrial usher protein with a FimD outer membrane domain (BMF81_00267), two SCPU domain-containing proteins (BMF81_00268 and BMF81_00270), and a pilus assembly protein PapD with a FimC domain (BMF81_00269). Closer homologs (BLASTp identity ≥80% and coverage ≥50%) can only be found in several cyanobacterial species including *Chrysosporum ovalisporum, Sphaerospermopsis kisseleviana* and *Dolichospermum compactum*, while remote homologs (BLASTp identity ≥60% and coverage ≥40%) mainly exist in Proteobacteria such as *Myxococcus, Collimonas* and *Rhodanobacter*, and several other cyanobacterial strains including *Trichormus* sp. NMC-1, *Calothrix* sp. NIES-2100, and *Alkalinema* sp. CACIAM 70d. Many of the shared clusters represent functionally well-described multi-copy gene families. For example, all three *Nodularia* harbor four identical copies of the *psbA* gene encoding the D1 protein of photosystem II, and 10 gene copies encoding proteins of the CAB/ELIP/HLIP superfamily. These proteins are supposed to support the highly robust photophysiology of these cyanobacteria when exposed to high irradiance and oxygen partial pressure at the Baltic Sea surface during summer (Kopf et al., 2015).

In addition to IS elements, another particular dynamic element in bacterial genome evolution are the CRISPR-Cas systems. These systems are frequently deleted and also become horizontally distributed (Godde and Bickerton, 2006). However, the draft status of previous genome analyses rendered the analysis of the *Nodularia spumigena* CRISPR apparatus with its many repeated sequence elements difficult. Our analysis unraveled two CRISPR-Cas repeat-spacer arrays on the chromosome of *Nodularia* UHCC 0039 (Figure S3). These encompass a total of 34 spacers. According to the presence of respective marker genes (Makarova et al., 2015), one major CRISPR-Cas system on the reverse strand of the chromosome, with its repeat-spacer region from 4035453–4037321 can be classified as subtype III-B. The intergenic regions (952013-952477, 4037653-4037322) between the two CRISPR loci and their upstream genes were compared against the CRISPRleader database (Alkhnbashi et al., 2016), assigning them to cluster F8B14. The *cas1* and c*as2* genes of this cassette on the reverse strand are separated from the other genes by a 16-gene insertion on the forward strand of the chromosome (Figure S3). The other repeat-spacer array from 952478–953238 lacks associated *cas* genes. Based on the conservation of the repeat sequences and secondary structures, both elements probably belong to the same system. Especially the conserved stem-loop structures (CTTTCCATAACCTCTT**CCCC**TAAC**GGGG**ATGGAAAC and GTTTTTCATAACCatTT**CCCC**gcAa**GGGG**AcGGAAAC) are relevant for recognition by the Cas6 maturation endonuclease (Reimann et al., 2017). The direct repeats of the two CRISPR arrays were moreover identical to their counterparts in *Nodularia* CCY9414, but all spacers differed from each other. Spacer sequences provide a memory of past infections, with the most recent additions represented by the most 5'-located spacers. Therefore, this finding indicates that the CRISPR systems in both strains were inherited from their joint ancestor, while all the spacers were acquired later, remaining as hallmarks of different infectious trajectories in the past. Transcripts were detected from both CRISPR loci in UHCC 0039 (Figure S3), suggesting the functionality of both CRISPR arrays.

### Conserved non-coding RNAs found in *Nodularia spumigena*

Increasing evidence has shown ncRNA is a major player in the regulatory systems of cyanobacteria and thus conserved non-coding RNAs were searched (Kopf and Hess, 2015). In this study, we identified 15 expressed intergenic regions and 24 antisense RNAs based on the transcriptome data of the here tested conditions (Table S5). Seven contained the DGR1 ncRNA elements (Table S5, highlighted in red) (Voß et al., 2013), which were widespread in the three *Nodularia* strains (75, 67, and 50 copies were found in CCY9414, UHCC0039, and CENA596 at an e-value cutoff of 1e-20), indicating that these DGR elements were active in the genome. The DGR1 sequences were highly conserved at the sequence level (Figure S4a) and at the secondary structure level (Figure S4b) even among three geographically separated *Nodularia* strains, suggesting that DGR1 was present in their common ancestor and evolved extremely slowly. In addition, the 9th strongly expressed intergenic regions (nsn009) in Table S5 encoded a long ncRNA that was only found in *Nostocales* based on a BLASTn search at an e-value cutoff of 1e-5. These homologs were conserved at the sequence level (Figure S4c) and in part at the secondary structure level, too (Figure S4d). The second hairpin structure in particular was highly conserved, and the sequence UGURCCUCC in the loop (highlighted by a green curve in Figure S4d) might function as a regulatory element.

### *Nodularia* UHCC 0039 growth in low and moderate salinities

**Figure 5:**
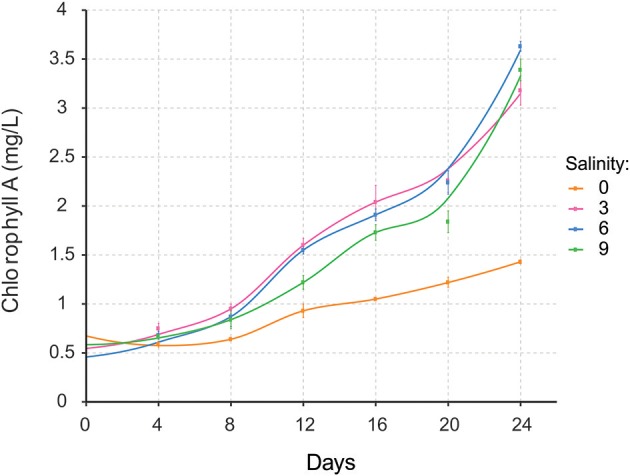
Growth of *Nodularia* UHCC 0039 at salinities of 0, 3, 6, and 9 g L^−1^ NaCl.

*Nodularia* UHCC 0039 growth was followed at four different salinities, 0, 3, 6, and 9 g L^−1^ NaCl by measuring chlorophyll *a* concentration (Figure 5). *Nodularia* UHCC 0039 survived in each of the tested salinities, but growth was heavily impaired at 0 g L^−1^ NaCl. No obvious growth differences were observed at 3, 6, or 9 g L^−1^ NaCl, suggesting flexibility of the *Nodularia* metabolism as long as some salt was present. Cell counting was applied to follow culture growth to exclude the effect of chlorosis on the estimation of cellular growth. Patterns of chlorophyll *a* and cell counts appeared to correlate well (Figure S5), and thus chlorophyll *a* concentration was later used for the normalization in our study.

### Transcriptome remodeling under unfavorable salinities

We applied a transcriptomic sequencing approach to study the transcriptional response of *Nodularia spumigena* to long-term low salinity (0 g L^−1^ NaCl), corresponding to general freshwater conditions. Transcriptional responses were analyzed at 0 g L^−1^ NaCl with reference to those at 6 g L^−1^ NaCl (analyzed at day 16), using a Log2 fold change (Log2FC) of ±1 and FDR < 0.01 as cut-off values: positive values (up-regulation) reflect increased expression at 0 g L^−1^ NaCl. The acclimation response of *Nodularia* UHCC 0039 to the lower salinity involved 302 up-regulated and 341 down-regulated genes (Table 1 and Tables S6, S7). The most up-regulated gene BMF81_04664 encodes Serpin B (serine protease inhibitor). RNA polymerase sigma factors have a key role in the acclimation to changes in environmental conditions. In addition to the vegetative major sigma factor SigA, there are five alternative group 2 sigma factors and five alternative group 3 sigma factors, which were classified by comparison against the set of well-characterized sigma factors from *Synechocystis* PCC 6803 (Figure S6). One group 2 (BMF81_04399) and one group 3 (BMF81_04729) sigma factor lack an obvious homolog in *Synechocystis* PCC 6803. The plasmid-encoded sigma factor BMF81_04729 is highly unique, which interestingly has no counterpart in the other two *Nodularia* strains and is fused to an unknown N-terminal region. Similar proteins exist only in four other cyanobacteria, *Oscillatoria acuminata* PCC 6304, *Tolypothrix bouteillei, Geminocystis herdmanii*, and *Trichodesmium erythraeum* IMS 101 (Figure S7). RNA polymerase group 2 sigma factors (BMF81_01360, BMF81_02088, BMF81_04429, and BMF81_00672 corresponding to SigB (Sll0306), SigD (Sll2012), SigC (Sll0184), and SigE (Sll1689) in the model *Synechocystis* PCC 6803, respectively (Figure S7), together with one group 3 sigma factor (BMF81_00786) were induced in zero salt whereas sigma factors BMF81_04399 and BMF81_04729 became repressed (Table 1 and Tables S6, S7). The fact that six out of 11 sigma factor genes displayed significant changed expression suggests a massive sigma factor-dependent remodeling of the transcriptome upon entering the zero salt condition.

**Table 1:** List of the selected differentially expressed genes of *Nodularia* UHCC 0039 while comparing low salinity (0 g L^−1^ NaCl) to normal salinity (6 g L^−1^ NaCl).

**GO term**	**locus_tag**	**Gene name**	**Product**	**Log2 FC**
**PHOTOSYNTHESIS AND ELECTRON TRANSPORT**				
GO:0009055	BMF81_00359		Cytochrome b6	1.66
GO:0009055	BMF81_01796	psbA	Photosystem II protein D1 2	1.28
GO:0005622	BMF81_04216	ccsB	Cytochrome c biogenesis protein	1.08
GO:0044424	BMF81_00668		Cytochrome c6	−1.58
GO:0043167	BMF81_00670		Cytochrome c6	−2.36
GO:0042651	BMF81_01995	psb27	Photosystem II lipoprotein	−1.02
GO:0034357	BMF81_02616	psbB/C	Chlorophyll a/b light-harvesting protein	−1.43
GO:0034357	BMF81_02622	pcb	Chlorophyll a/b light-harvesting protein	−2.16
GO:0034357	BMF81_02857	cpcD	Photosystem I reaction center XII	−1.15
GO:0034357	BMF81_02858	cpcD	Photosystem I reaction center XII	−1.32
**CELL MEMBRANE AND CELL DIVISION**				
GO:0071840	BMF81_00264		N-acetylmuramoyl-L-alanine amidase	2.6
GO:0071840	BMF81_03893		N-acetylmuramoyl-L-alanine amidase	1.67
GO:0071704	BMF81_02030	lytC	Peptidoglycan-N-acetylglucosamine deacetylase	1.27
GO:0071704	BMF81_03481	murB	UDP-N-acetylenolpyruvoylglucosamine reductase	1.45
GO:0071704	BMF81_03482	murC	UDP-N-acetylmuramate L-alanine ligase	1.28
**TRANSCRITPION AND TRANSLATION**				
GO:0016987	BMF81_01360	sigB	RNA polymerase sigma factor	2.08
GO:0016987	BMF81_00786	sigG	RNA polymerase sigma factor	1.25
GO:0016987	BMF81_02088	sigD	RNA polymerase sigma factor	1.3
GO:0016987	BMF81_04429	sigC	RNA polymerase sigma factor	1.25
GO:0016987	BMF81_00672	sigE	RNA polymerase sigma factor	1.12
GO:0016987	BMF81_04399		RNA polymerase sigma factor	−1.7
GO:0016987	BMF81_04729		RNA polymerase sigma factor	−1.02
GO:0043226	BMF81_03163	rimO	Ribosomal protein S12 methylthiotransferase	1.22
GO:0034062	BMF81_02138		DNA-directed RNA polymerase subunit beta	1.08
GO:0034062	BMF81_02139		DNA-directed RNA polymerase subunit beta	1.16
**CHAPERONES**				
GO:0005515	BMF81_04277	dnaJ	Chaperone protein	1.33
GO:0043167	BMF81_04014	clpB1	ATP-dependent chaperon	1.37
GO:0043167	BMF81_04028	dnaK	Chaperone protein	1.29
GO:0005515	BMF81_01878	dnaJ	Chaperone protein	1.21
GO:0051082	BMF81_00304		Chaperone protein dnaK2	1.99
GO:0051082	BMF81_00306		Chaperone protein dnaK2	1.74

**Figure 6:**
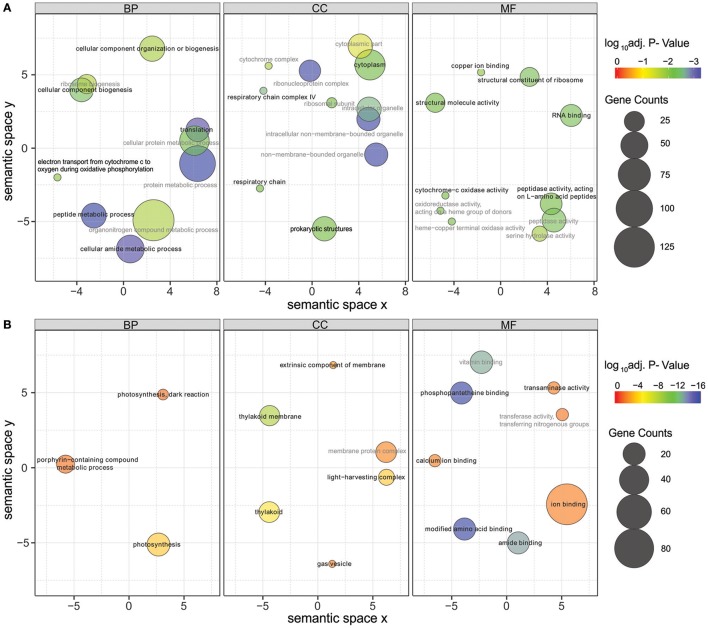
Gene Ontology (GO) enrichment analysis of the up-regulated **(A)** and down-regulated **(B)** genes in low salinity (0 g L^−1^ NaCl) compared to normal condition (6 g L^−1^ NaCl). Circle size is proportional to the number of differentially expressed genes (FDR ≤ 0.01) assigned to each GO term and the color indicates the log_10_ adjusted *p*-value under hypergeometric test. Only GO terms with an adjusted *p*-value < 0.05 are shown. Pairwise semantic similarities of enriched GO terms were calculated using REVIGO with the SimRel method (Supek et al., 2011). GO terms with a dispensability <0.15 were taken as representatives and were highlighted in black color and bold font, the remaining GO terms were taken as redundant and were shown in gray color. BP, biological process; CC, cellular components; MF, molecular function.

Significantly differentially expressed genes (FDR < 0.01) were further used in gene ontology (GO) enrichment analysis to identify over- and under-represented GO terms (Figure 6). Several differentially expressed genes were classified to participate in cellular component organization or biogenesis (GO:0071840; e.g., BMF81_00264 and BMF81_03893). The demand for cell wall reorganization was further evidenced by up-regulation of genes related to peptidoglycan synthesis (GO:0071704: BMF81_03481-2). Photosynthesis and electron transport functions were heavily influenced by lowered salinity. Genes in the GO terms thylakoid (GO:0042651), photosynthetic membranes (GO:0034357), and phycobilisome (GO:0030089) together with photosynthesis (GO:0015979) and chlorophyll metabolic processes (GO:0015994) were significantly down-regulated. However, electron carrier activity (GO:0009055) and respiratory chain complex (GO0098803 and GO0070469) exhibited increased gene expression, showing the reorganized balance between the photosynthetic complex and respiratory chain. Transcription (GO:0034062, e.g., RNA polymerases BMF81_02138-9), translation (GO:0005840, e.g., ribosomal protein S12 methylthiotransferase BMF81_03163), and protein metabolism (GO:0006518, GO:0019538, and GO:0043043) were induced in low salinity, further demonstrating the high demand for structural and metabolic reorganization of the cell. Chaperones, molecules with an important role in protein folding, protection, and repair under stress conditions, were also highly up-regulated (BMF81_04014 and 04028).

Trehalose and sucrose are the major compatible solutes participating in the protection of the cellular component under increased salinity in freshwater cyanobacteria (Klähn and Hagemann, 2011). *Nodularia* UHCC 0039 possesses sucrose synthases genes along with trehalose synthase genes, similar to *Nodularia spumigena* CCY9414 (Voß et al., 2013), but these genes were not differentially expressed in our current study.

AntiSMASH was used to annotate the gene clusters of naturally produced biosynthetic gene clusters. Results showed that the genome of *Nodularia* UHCC 0039 harbors five known and two unknown gene clusters for the synthesis of natural products (Table S8). The known metabolites are nodularin, aeruginosin, nodulapeptin, spumigin, and heterocyst glycolipid. Of the known biologically active peptides, nodularin concentration was followed every fourth day (Figure S8). Nodularin concentrations increased over time in both low and high salinities, probably due to the accumulation of continuously produced toxins into cultures, while no differences were detected in nodularin concentrations or in the expression of genes within the nodularin gene cluster between low and moderate salinities. Interestingly, expression of the large genetic region containing 33 genes (BMF81_00493 and BMF81_00528) was heavily repressed (Log_2_FC < −1). This particular region includes hybrid nonribosomal peptide synthetase and polyketide synthase gene clusters (BMF81_00493-507; BMF81_00524-526), but the products are unknown.

## Discussion

Species diversity in brackish water ecosystems is usually relatively narrow because most organisms have adapted to life in either marine or freshwater environment (Telesh et al., 2013). However, cyanobacteria diversity is high in the brackish water Baltic Sea (Celepli et al., 2017) and, hence, the underlying phenomena should be studied in much greater detail. In our study, *Nodularia* UHCC 0039 was found to proliferate equally well in all tested salinities above and including 3 g L^−1^ NaCl, whereas growth was severely hampered in freshwater conditions (0 g L^−1^ NaCl). Our findings agree well with previous studies carried out in the Baltic Sea, where salt concentration plays an important role in the abundance and intensity of *Nodularia spumigena* and *Dolichospermum* in the blooms (Lehtimäki et al., 1997; Stal et al., 2003; Brutemark et al., 2015). *Nodularia* dominates in more saline parts (Halinen et al., 2007; Sivonen et al., 2007). The optimal salt concentration for Baltic Sea *Nodularia spumigena* CCY9414 growth was previously found to be 12.5 g L^−1^ NaCl, which is the salinity prevailing in the southern regions of the Baltic Sea (Möke et al., 2013).

### Comparative genomics

*Nodularia spumigena* is filamentous, capable of fixing atmospheric nitrogen and producing toxins, is buoyant, and forms akinetes in unfavorable conditions (Stal et al., 2003; Castenholz, 2015). The observed abundance of *Nodularia spumigena* in the Baltic Sea is thus the consequence of ecological niche adaptation, which can be obtained at the genome and gene regulation levels. The genome size of *Nodularia* UHCC 0039 was 5.39 Mb, which matches its previously sequenced counterparts *Nodularia* CCY9414 (5.46 Mb, Voß et al., 2013) and *Nodularia spumigena* CENA596 (5.2 Mb; Popin et al., 2016). In the phylogenomic tree of *Nostocales, Nodularia spumigena* encompasses one branch, which corresponded with the cluster Id distinguished in the average nucleotide and amino acid identity analyses. Our phylogenomics analysis further demonstrates the close relationship of *Nodularia spumigena* strains and highlights the joint evolutionary origin of Baltic Sea *Nodularia spumigena* strains (Laamanen et al., 2001; Lyra et al., 2005).

Syntenic regions were identified within the Baltic Sea *Nodularia spumigena* UHCC 0039 and CCY9414, but plasticity was also ubiquitous between UHCC 0039 and CENA596. Increased numbers of IS elements in the genomes of the Baltic Sea *Nodularia spumigena* compared to *Nodularia spumigena* CENA596 explain brackish water adaptation, as IS elements are a great source of genetic rearrangement and environmental adaptation (Frangeul et al., 2008). Phage-related horizontal gene transfer is another mechanism accelerating the evolution of bacteria and enabling them to adapt quickly in changing environments (Leitet et al., 2006; Paul, 2008; Shi and Falkowski, 2008). Although we found no phage remnants in the genome of *Nodularia* UHCC 0039, there is evidence that lytic bacteriophages affect *Nodularia spumigena*, and Baltic Sea cyanobacteria are thus highly exposed to viruses (Cairns et al., 2017; Coloma et al., 2017). To defend against viruses and other foreign DNA, bacteria have evolved several mechanisms, such as restriction endonucleases and CRISPR-Cas systems, which are found also within cyanobacteria (Hein et al., 2013; Scholz et al., 2013). For example, all studied *Microcystis aeruginosa* strains carried at least one CRISPR loci in their genomes, and 10 CRISPR-Cas systems were identified from the genome of *Microcystis aeruginosa* PCC 9717 (Yang et al., 2015). Here, two CRISPR loci with almost identical repeats were identified in the genome of *Nodularia* UHCC 0039. One of these loci lacked *cas* genes, which suggests that the two belong to the same CRISPR array.

### Transcriptome adaptation to a new environment

Genomes of cyanobacteria are highly dynamic and the induction of either general or stress-specific genes and regulatory systems enable them to proliferate in a changing environment. Transcriptome modifications that acclimate to elevated salt concentration are induced rapidly (Billis et al., 2014), but complete acclimation of metabolic processes resulting in the inhibition of cell division requires a longer time period (Qiao et al., 2013; Al-Hosani et al., 2015). For example, work on *Synechocystis* sp. strain PCC 6803 showed that the number of differentially expressed genes peaked after 30 min of salt shock and the majority of these genes returned back to the control level after acclimation for 24 h (Marin et al., 2004). Relying on the number of differentially expressed genes found after 16 days of incubation in various salt concentrations, we assume that the reconstruction of a transcriptional pattern remained vivid long enough to minimize the harm of unfavorable salt conditions, fine-tune the metabolic patterns, and replace the damaged proteins. Specific sigma factors play crucial roles in the regulation of gene expression and acclimation to a new environment. Especially group 2 sigma factors, such as up-regulated SigB, SigC, and SigD homologs in *Synechocystis* sp. PCC 6803 are important in environmental acclimation, being involved in high salt, response to heat stress, nitrogen starvation, and further environmental perturbations (Marin et al., 2004; Tuominen et al., 2005; Singh et al., 2006; Nikkinen et al., 2012; Antal et al., 2016). Moreover, we observed a matching induction of another sigma factor, BMF81_00672, which corresponds to SigE, (Sll1689) of the model *Synechocystis* sp. PCC 6803 (cf. Figure S4), which up-regulates the expression of genes encoding proteins involved in sugar catabolism (Osanai et al., 2005, 2011). Interestingly, decreased expression was found in one group 2 (BMF81_04399) and one group 3 (BMF81_04729) sigma factor, which lacked direct homologs in *Synechocystis* PCC 6803. Hence, these alternative sigma factors appear to have a role in the prevailing brackish water conditions.

Accumulation and production of compatible solutes together with ion exchange over the cell membrane are major salt-stress acclimation strategies for minimizing the effects of increased intracellular ion concentrations (Hagemann, 2011). Low halotolerant strains are able to produce the compatible solutes trehalose and sucrose (Klähn and Hagemann, 2011). In our study, none of the genes previously specifically known for salt stress appeared to remain up-regulated for 16 days. In turn, the metatranscriptomics study of the Baltic Sea showed that transcripts of trehalose synthase were not found, whereas transcripts of sucrose synthase were present at a salinity of <20 practical salinity unit (psu) (Celepli et al., 2017). These results also matched the observations by Voß et al. (2013) on the expression of these genes in *Nodularia* CCY9414. In addition, accumulation of compatible solutes was measured in *Nodularia* CCY9414, and sucrose appeared to be the major compatible solute (Möke et al., 2013). The possible role of trehalose as a compatible solute in brackish water ecosystems is thus questionable and needs more investigations.

In general, salt shock inhibits the proper functioning of several proteins and metabolic processes, such as photosynthesis, central metabolism, and cellular growth, and the high demand for the reconstruction of cellular metabolism is obvious (Marin et al., 2004; Allakhverdiev and Murata, 2008; Billis et al., 2014; Rai et al., 2014; Al-Hosani et al., 2015). Chaperones, molecules with an important role in protein folding, protection, and repair under stress conditions, were highly up-regulated in our study. Their role in maintaining protein integrity under low and high salt stress was previously described (Fulda et al., 2006; Al-Hosani et al., 2015). The cell wall is the first part encountering a new environment, and thus cell wall structure and functioning have a crucial role in adaptation. In our study, the induction of the respective genes pointed at the reconstruction of cell wall structures in unfavorable low salt concentrations. Cell wall structure and composition have previously been shown to change drastically in increased salt concentrations (Poolman et al., 2004; Huang et al., 2006) and a similarly reorganized cell wall is also needed in unsuitable low salinity conditions (our study).

### Natural products in unfavorable salinities

Cyanobacterial genomes harbor a great variation of gene clusters for the production of biologically active natural products, of which toxins, such as nodularin and microcystin, are the most studied molecules due to their harmfulness against mammals (Welker and von Döhren, 2006). Similarly to *Nodularia spumigena* CCY9414, *Nodularia* UHCC 0039 harbors five gene clusters for chemically characterized bioactive compounds, including the nodularin toxin (Fewer et al., 2013; Voß et al., 2013). Nodularin concentration was previously reported to decrease in extreme salinities (Lehtimäki et al., 1997), but here the tested salinity range was most probably too narrow and the nodularin concentration remained constant in all conditions. The drastically down-regulated region encompassing genes BMF81_00439 to BMF81_00526 shows high similarity to the siderophore synthase gene cluster in *Agrobacterium tumefaciens* C58 (Rondon et al., 2004), but the product in cyanobacteria has not been identified. However, in unfavorable conditions, where cell growth and metabolism are heavily repressed, expression of this gene cluster appeared redundant and thus transcription was hindered.

## Author contributions

JT and KS designed and executed the experiment, and prepared the samples for sequencing. SH and WH designed the computational analysis pipeline, performed the bioinformatics analyses and prepared the figures. MW analyzed toxin samples. JT, SH, MW, WH, and KS wrote the manuscript.

### Conflict of interest statement

The authors declare that the research was conducted in the absence of any commercial or financial relationships that could be construed as a potential conflict of interest.
