# Supplementary material for: Comparative Genomics of the Baltic Sea Toxic Cyanobacteria Nodularia spumigena UHCC 0039 and Its Response to Varying Salinity
Source: Front Microbiol. 2018 Mar 8;9:356. doi: 10.3389/fmicb.2018.00356 (PMC5853447; doi:10.3389/fmicb.2018.00356)
Supplement: Supplementary file 1 [file Image1.PDF]

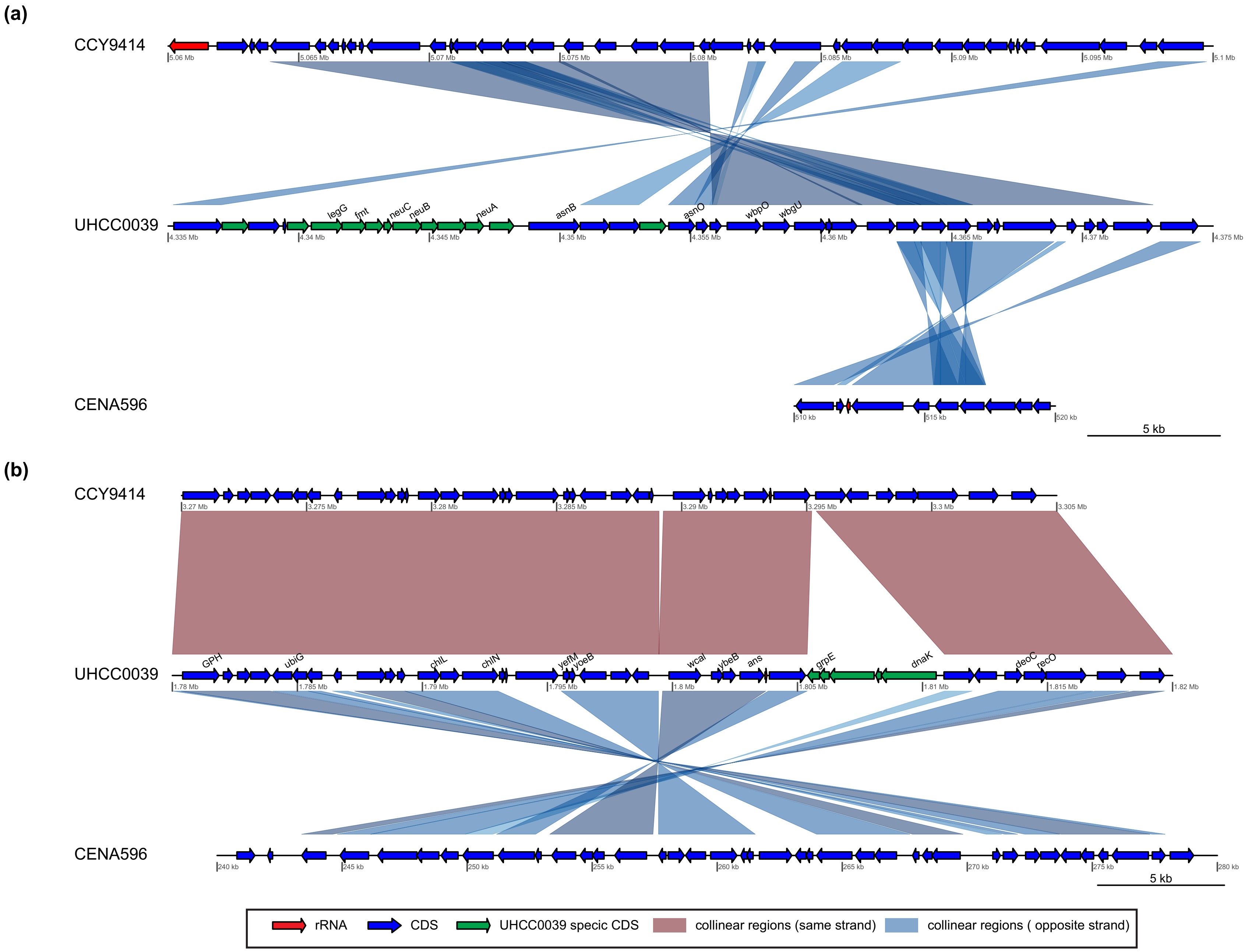

Figure S1 Selected insertion blocks of UHCC 0039 compared to the other *Nodularia* strains. Gene cassettes encoding surface-modifying enzymes (a) and chaperons, co-chaperons and peptidases (b).
