# Supplementary material for: Comparative Genomics of the Baltic Sea Toxic Cyanobacteria Nodularia spumigena UHCC 0039 and Its Response to Varying Salinity
Source: Front Microbiol. 2018 Mar 8;9:356. doi: 10.3389/fmicb.2018.00356 (PMC5853447; doi:10.3389/fmicb.2018.00356)
Supplement: Supplementary file 3 [file Image3.PDF]

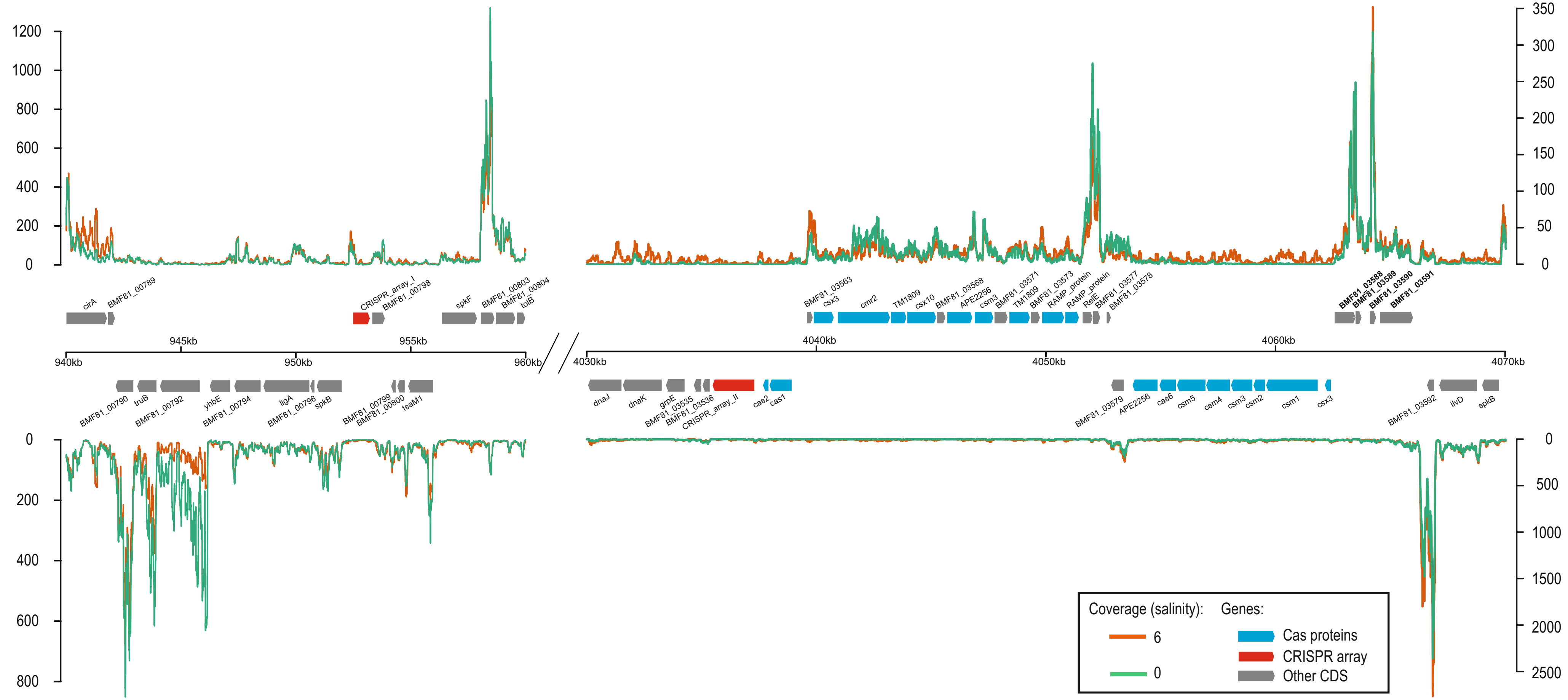

Figure S3 CRISPR-Cas system and transcriptome coverage. CRISPR array was highlighted in red, Cas genes were highlighted in light blue. The average coverages for the three replicates in each condition were shown. Upper panel represents transcriptional coverage in forward strand and lower panel represents transcriptional coverage in reverse strand.
