# Supplementary material for: Comparative Genomics of the Baltic Sea Toxic Cyanobacteria Nodularia spumigena UHCC 0039 and Its Response to Varying Salinity
Source: Front Microbiol. 2018 Mar 8;9:356. doi: 10.3389/fmicb.2018.00356 (PMC5853447; doi:10.3389/fmicb.2018.00356)
Supplement: Supplementary file 6 [file Image6.PDF]

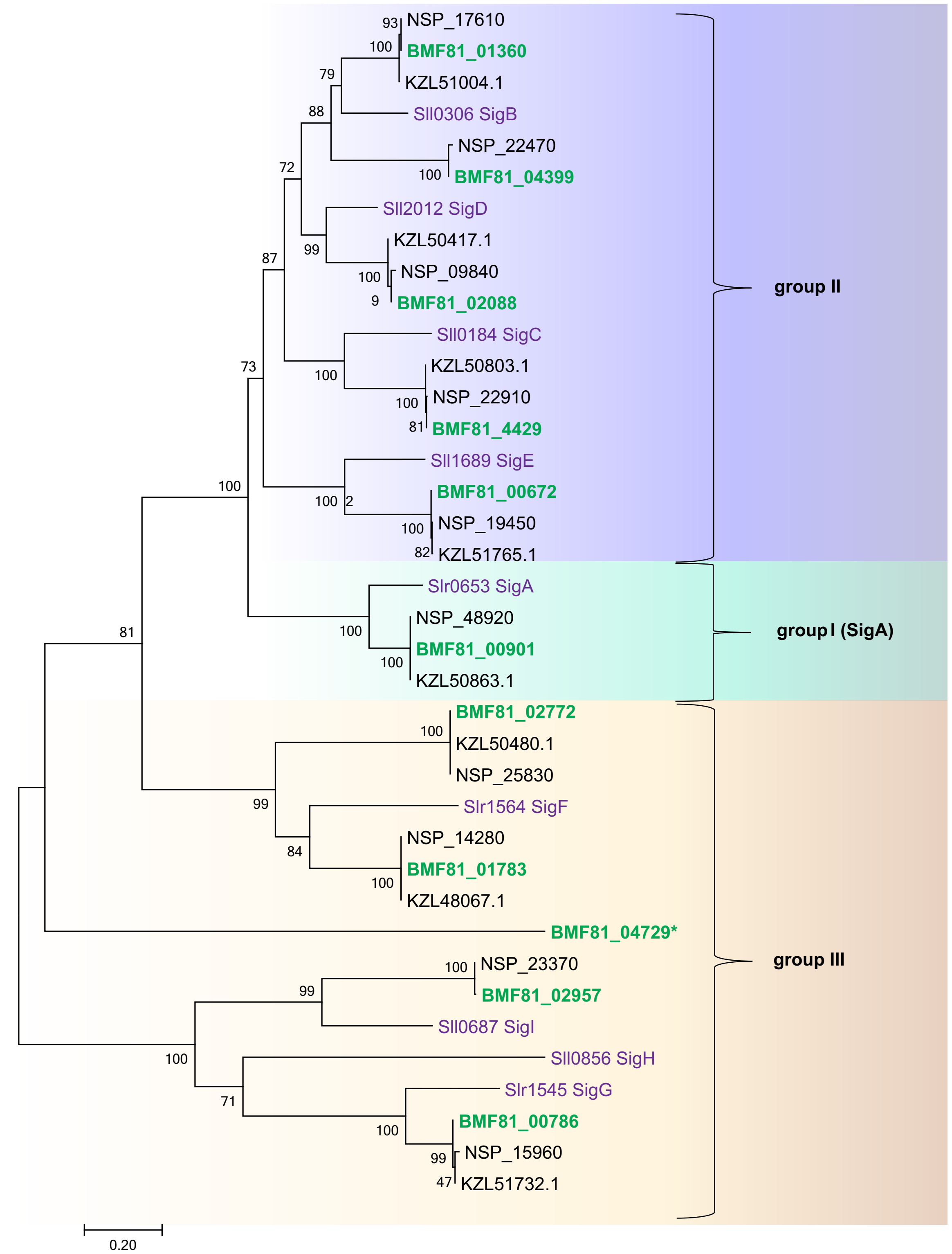

Figure S6 Phylogenetic relationships amongst sigma factors from *Nodularia* UHCC 0039 (highlighted by green boldface letters) compared with those from the other two sequenced *Nodularia* (sequences from *N. spumigena* CCY9914 start with the prefix NSP and from strain CENA596 with KZL). The putative orthologous proteins can also be found in Table S1. The vegetative major sigma factor SigA, the four alternative group 2 sigma factors and four alternative group 3 sigma factors from *Synechocystis* PCC 6803 were included for orientation (in dark blue). The unique, plasmid-encoded sigma factor BMF81_04729 is labelled by an asterisk, for its sequence alignment with four similarly structured sigma factors from other cyanobacteria see Figure S5.

The evolutionary history was inferred using the Minimum Evolution method (Rzhetsky and Nei, 1992) as implemented in MEGA 7 (Kumar et al., 2016).The optimal tree with the sum of branch length = 9.596226 is shown. The percentage of replicate trees in which the associated taxa clustered together in the bootstrap test (1000 replicates) are shown next to the branches. The tree is drawn to scale and distances are in the units of the number of amino acid substitutions per site as indicated by the scale bar. The analysis involved 38 amino acid sequences with a total of 478 positions in the final dataset.
