# Supplementary material for: Comparative Genomics of the Baltic Sea Toxic Cyanobacteria Nodularia spumigena UHCC 0039 and Its Response to Varying Salinity
Source: Front Microbiol. 2018 Mar 8;9:356. doi: 10.3389/fmicb.2018.00356 (PMC5853447; doi:10.3389/fmicb.2018.00356)
Supplement: Supplementary file 7 [file Image7.PDF]

```
                                      10        20        30        40        50        60        70        80        90       100
                              ....|....|....|....|....|....|....|....|....|....|....|....|....|....|....|....|....|....|....|....|
N.spu._UHCC0039_04729         --MQTLSWQDLENIKSPESISGLFDKLGYNTCCEPVDVNDLELSQTNQGIIDTVYLIANQGESDLQVFLFQLNPAYWANENNAIARLKSIAKSLCKRPSY
O.acuminata_WP_015149509.1    MVYRHIHIEDLEHIKDALSVAVLFESLGYDADCDPLEVSDLQLSPSNTEAIKTAYLIANQEKGTLQVFLLELEPKSWSSNAGVIVRLTAIAKNLCQRDTD
T.bouteillei_WP_050046850.1   MSDRLLHLDDVKNIRKPEQVAAIFQKLGYKAICELLDVTDLELSKSSTQAVNHVYLIANQGNAELQVLLFQLKPSEWLSLDAVSYRMQAIAKSLCQRLSY
G.herdmanii_WP_017293552.1    MIKEELKVRDLELISSSEDIADLFKKLNYTIINEPIDIEKFTFSSTNYYAIKKAYLIASEKNSDLQVVLFELDYSTWVSSSIYLPRIESISKQISNRSSD
T.erythraeum_WP_052845755.1   MIGHTLELDDLKKISSPKKIVALFEKLGFNFQIEHLDIEQLELPSYCRETIDNTYKIYNPNQEELQVFLLELRQEKFKTNYKEQERIITISQGILRNKNN

                                     110       120       130       140       150       160       170       180       190       200
                              ....|....|....|....|....|....|....|....|....|....|....|....|....|....|....|....|....|....|....xxxxxxxxxxx
N.spu._UHCC0039_04729         FLIVGMISYQKLLLVSPVRSFNAQMELEIGTRQIPIDLKSASLYDLHLLQRIAANNLEPKSLYQTQHRVFLDAQRLGIKAEEKKANRDKDTVRDYLVAIG
O.acuminata_WP_015149509.1    ILIVATVNYQQLVLISPFQTLDQQLNLKLSIAKMFINVKNPSFYDLNQLEKIAAHSLKPSQLYQVQHQTLKQVR------EEKRQPISTDLVQCYLREIG
T.bouteillei_WP_050046850.1   FLLLCTRDYSQLMLVSPRMSFDARMNFRLNINKYFINTADPSYHDLNRLEKIAAFSLEPQTLHRIQHEALRFREL---QKQYET----QDSIRWYLQQIG
G.herdmanii_WP_017293552.1    FLVIGTVAYKNLVFVKSSGKFDENYNFRVEINTTVVNLQEVDLSTLHFIKSLTITENDLNLLNKRQEIAIKNIN----REAKKRNQLSTDSLGFYLQEIG
T.erythraeum_WP_052845755.1   FLLIATKKYNKLIIVAPHR--RENQNIKISIKWWLIDCQNPNYYDTYWLENVAINNCEPTEELTNKHNSEGSYDP---DIEPPTGTFTEDSIQLYYKQVN

                                     210       220       230       240       250       260       270       280       290       300
                              xxxxxxxxxxxxxxxxx..|....|....|....|....|....|....|....|....|....|..ooooooooooooooooooooooooooooooooo
N.spu._UHCC0039_04729         KIKLLKPEDEILLSRKIVRSSELNESKKILRELLDREPTNREWADINNLSISRLYDELAVGYAAKNKLVESNLRLVVSIAKKYQNRGLDLLDLIQEGNLG
O.acuminata_WP_015149509.1    RIPLLKREDEISLAQQVQRWIQLEQGKQSLNSKLHREPTRSEWANATGLSLSQLEDAISKGRKAQKSLVYSNLRLVVSIAKIYQHRGLELLDLIQHGNEG
T.bouteillei_WP_050046850.1   RIKLLKASEEIFLARQVAQLEALENIRQRLQKQHQRNPQDEELALEFGISVSLLRERIANGRFAKNKLIQANLRLVVSITKNYINRGVDFLDLIQEGNLG
G.herdmanii_WP_017293552.1    KIPLLSQEQEIILSKQIQELVLLEKKRETLKKQLSQEPTTKEWAEFVKISYKHLVIKISQGEKAKRKMVEANLRLVVSIAKKYNSPNLDLLDLIQEGSIG
T.erythraeum_WP_052845755.1   SFARLHPEEEIHLAHQIDGLLALEKIYEKLEEKLNRSPNEIEWATEAKMSKWELCDRLEQGRRAKNQMVTANLRLVTSVAKRYQNRGLDFQDLIQEGVIG

                                     310       320       330       340       350       360       370
                              ooooooooo|ooo.oooooooooooooooooo..|....|....|....|....|....|....|....|.
N.spu._UHCC0039_04729         LIRAAEKFDPTKGYRFSTYATCWIRQAITRDIYNSSRLIRIPVHFWDRLREIKKAQREILEQGLSYPSIKDIAEWH
O.acuminata_WP_015149509.1    LIKATEKFDPIKGYKFSTYATHWIRQHITRGLCNESRIIRLPVHIQESLSKVKKTTRHLTLKLEHIPSLEKIAVEL +250 aa
T.bouteillei_WP_050046850.1   LIKAVEKFDYTKGYQLSTYATWWIRQGIRRAINNQSRTIRLPVHLWEKISLIKKTTKQLSQEIGRLPKQKEIAASL  +96 aa
G.herdmanii_WP_017293552.1    LIRGTEKFDPTKGYRFSTYAYWWIRQAITRYLCNYNRVIRLPIHLWEYQQQIKKAYRDLIAKNQIISTSNIIKYLG  +38 aa
T.erythraeum_WP_052845755.1   LLRATEKFDHTKGYKFSTYGIWWIRQAITRAICDYSRIVRLPVYLYETISQIKKIVKQISIECSPLT-AEEVATRM +124 aa
```

Figure S7 Alignment of plasmid-encoded sigma factor BMF81_04729 with four similarly structured sigma factors from other cyanobacteria.
