# Supplementary material for: Comparative Genomics of the Baltic Sea Toxic Cyanobacteria Nodularia spumigena UHCC 0039 and Its Response to Varying Salinity
Source: Front Microbiol. 2018 Mar 8;9:356. doi: 10.3389/fmicb.2018.00356 (PMC5853447; doi:10.3389/fmicb.2018.00356)
Supplement: Supplementary file 8 [file Image8.PDF]

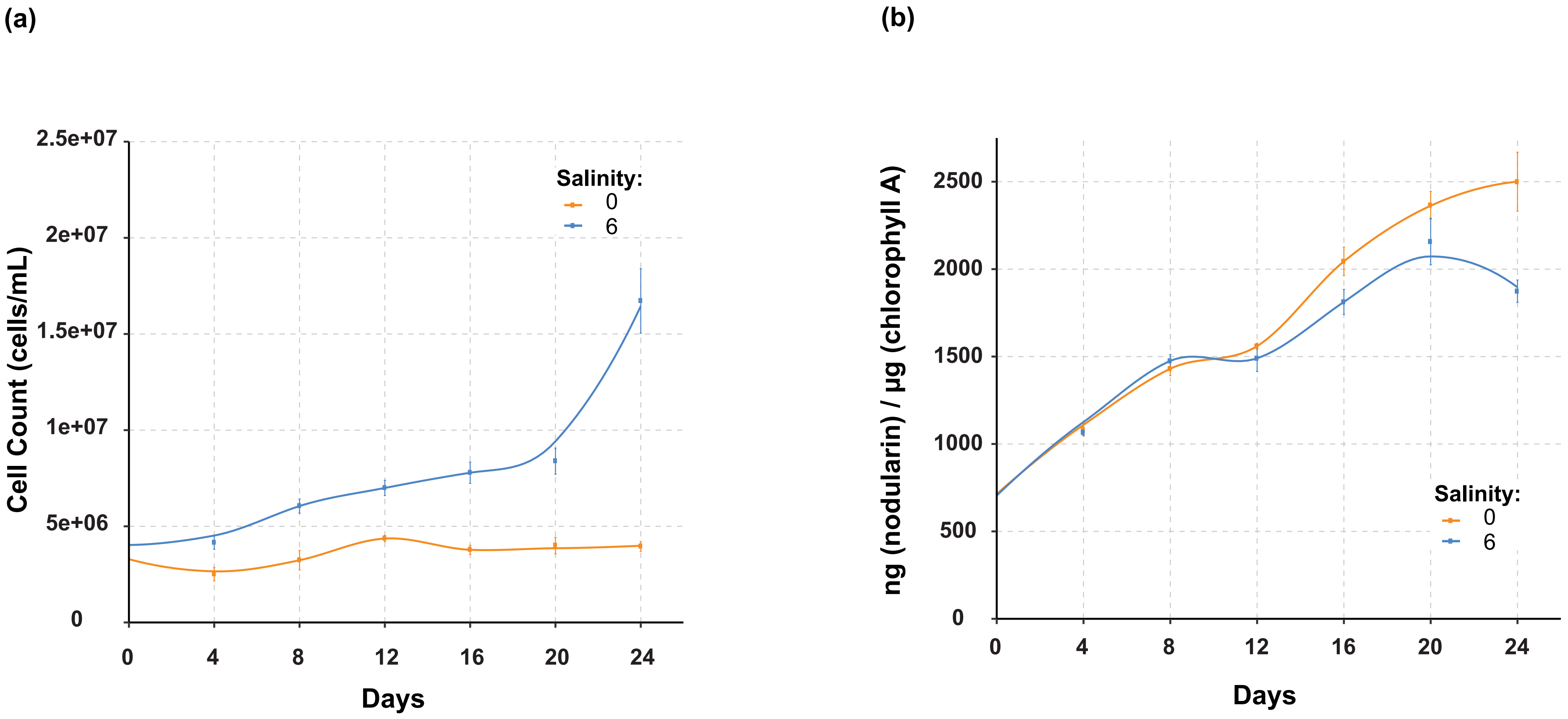

Figure S8 Cell counts (a) and normalized nodularin concentration (b) of *Nodularia* UHCC 0039 in low and moderate salinities.
