# Supplementary material for: Comparative Genomics of the Baltic Sea Toxic Cyanobacteria Nodularia spumigena UHCC 0039 and Its Response to Varying Salinity
Source: Front Microbiol. 2018 Mar 8;9:356. doi: 10.3389/fmicb.2018.00356 (PMC5853447; doi:10.3389/fmicb.2018.00356)
Supplement: Supplementary file 17 [file DataSheet1.DOCX]

**Supporting information**

**Supporting figures**

**Figure S1** Selected insertion blocks of UHCC 0039 compared to the other *Nodularia* strains. Gene cassettes encoding surface-modifying enzymes (a) and chaperons, co-chaperons and peptidases (b).

**Figure S2** Number of identified IS elements in *Nodularia* (a) and PCA analysis of the IS families among *Nodularia* (b).

**Figure S3** CRISPR-Cas system and transcriptome coverage. CRISPR array was highlighted in red, Cas genes were highlighted in light blue. The average coverages for the three replicates in each condition were shown. Upper panel represents transcriptional coverage in forward strand and lower panel represents transcriptional coverage in reverse strand.

**Figure S4** Selected expressed non-coding RNAs and the conserved secondary structures. Sequence alignment and secondary structure of DGR1 (a, b) and a conserved ncRNA nss009 (c, d).

**Figure S5** Correlation between chlorophyll A and cell counts. For each condition, a linear regression line and the coefficient of determination was calculated.

**Figure S6** Phylogenetic relationships amongst sigma factors from *Nodularia* UHCC 0039 (highlighted by green boldface letters) compared with those from the other two sequenced *Nodularia* (sequences from *N. spumigena* CCY9914 start with the prefix NSP and from strain CENA596 with KZL). The putative orthologous proteins can also be found in Table S1. The vegetative major sigma factor SigA, the four alternative group 2 sigma factors and four alternative group 3 sigma factors from *Synechocystis* PCC 6803 were included for orientation (in dark blue). The unique, plasmid-encoded sigma factor BMF81_04729 is labelled by an asterisk, for its sequence alignment with four similarly structured sigma factors from other cyanobacteria see Figure S5.

The evolutionary history was inferred using the Minimum Evolution method (Rzhetsky and Nei, 1992) as implemented in MEGA 7 (Kumar et al., 2016).The optimal tree with the sum of branch length = 9.596226 is shown. The percentage of replicate trees in which the associated taxa clustered together in the bootstrap test (1000 replicates) are shown next to the branches. The tree is drawn to scale and distances are in the units of the number of amino acid substitutions per site as indicated by the scale bar. The analysis involved 38 amino acid sequences with a total of 478 positions in the final dataset.

**Figure S7** Alignment of plasmid-encoded sigma factor BMF81_04729 with four similarly structured sigma factors from other cyanobacteria.

**Figure S8** Cell counts (a) and normalized nodularin concentration (b) of *Nodularia* UHCC 0039 in low and moderate salinities.

**Supporting Tables**

**Supplementary Table S1** General information of the genomes and assemblies used in this study.

**Supplementary Table S2** Pairwise BLASTn based genome-wide average nucleotide identity matrix.

**Supplementary Table S3** Pairwise average amino acid identity matrix.

**Supplementary Table S4** *Nodularia spumigena* orthologous gene clusters and genes present only in one or two genomes.

**Supplementary Table S5** Intergenic regions and antisense-RNA identified from *Nodularia* UHCC 0039.

**Supplementary Table S6** List of the upregulated genes (Log_2_FC >1) of *Nodularia* UHCC 0039 based on edgeR test.

**Supplementary Table S7** List of the downregulated genes (Log_2_FC <-1) of *Nodularia* UHCC 0039 edgeR test.

**Supplementary Table S8** Gene clusters responsible of the production of known and putative natural products in *Nodularia* UHCC 0039.

**Supporting materials and methods**

*Genome annotation*

Genome of *Nodularia* UHCC 0039 was annotated using Prokka v1.12 (Seemann, 2014) and ncRNA by querying rfam database (Griffiths-Jones et al., 2003) using cmscan from Infernal (Nawrocki et al., 2009). InterProScan 5 (Cock et al., 2013) was used to polish the protein annotation, and followed by manually curation in genome browser Artemis (Carver et al., 2012). BlastKOALA v2.1 (Kanehisa et al., 2016) was used to annotate KEGG orthology (KO). COG orthology was annotated by querying NCBI Conserved Domain Database (CDD) (Marchler-Bauer et al., 2015) with e-value cutoff 1e-5 using RPSBLAST 2.2.31+ (Camacho et al., 2009). BLAST2GO (Conesa et al., 2005) was used to annotate the GO term. Putative secondary metabolites and biosynthetic gene clusters were predicted using antiSMASH online server (Weber et al., 2015). Genome and plasmid sequences were scanned against the CRISPRdb using CRISPRfinder (Grissa et al., 2007) to annotate CRISPR loci. IS elements were annotated using ISsaga webserver (Varani et al., 2011).

*Phylogenetic inference*

A maximum-likelihood phylogenomic tree was constructed by the concatenation of 31 universal marker genes (Wu and Eisen, 2008). In brief, the profile of these marker genes (Wu and Eisen, 2008) were scanned against open reading frames (ORFs) of reference genome sequences using HMMER v3.1b2 (Eddy, 1998), detected ORFs were aligned using muscle v3.6 (Edgar, 2004) with default parameters, then the alignments were trimmed by trimAl v1.2 with parameters “-gt=0.9, -cons=60, -w=3” to remove gap positions. For each reference genome, the trimmed alignments of marker proteins were concatenated into a super matrix sequentially, then partitioning schemes and best substitution models were estimated using PartitionFinder v1.1.1 (Lanfear et al*.*, 2012), and a maximum likelihood tree was estimated by RAxML v8.1.20 (Stamatakis, 2014) based on selected partitions and substitution models, with 1000 rapid bootstrap searches. *Candidatus* Pelagibacter Ubique HTCC 1062 was selected as an outgroup to root the phylogenomic tree.

In total, 28 selected complete and draft genomes of cyanobacteria were included in the comparative genomics study. The average genome-wide nucleotide identity (ANI) was computed with ANIb algorithm which cuts the genome into pieces of 1020 nt fragments, and then pairwise blastn was done using these fragments as inputs. The detailed explaination of this algorithm can be found in the Jspecies package (Richter and Rosselló-Móra, 2009). The specific program used in this study was pyani v0.2.1 (https://github.com/widdowquinn/pyani), which is a python implementation of the algorithm. The AAI was computed on protein level. Pairwise orthologous genes among genomes were determined using the reciprocal best hits algorithm, then the AAI was calculated as the mean amino acid identity using CompareM v0.0.18. The program used in this study is available at https://github.com/dparks1134/CompareM. Orthologous groups were inferred using OrthoVenn (Wang et al., 2015) with a maximum e-value cutoff of 1e-5 and an inflation index of 1.5 for MCL clustering (Li et al., 2003). Multiple genome alignments were performed using progressive Mauve alignment algorithm with Mauve v2.4 (Darling et al., 2004). Local synteny blocks were visualized using genoPlotR v0.8.4 package (Guy et al*.*, 2010).

For the synteny analysis, the scaffold of CCY9414 was split into contigs at assembly gaps. Contigs of CCY9414 and CENA596 were aligned and reordered according to UHCC0039 genome using progressiveMauve (v2015-02-13) with default parameters (Darling et al., 2010). The reordered contigs were joined into artificial scaffolds with 100 Ns in between of each pair of neighboring contigs. Similarities between the three genomes were compared using BlastN with an E-value cutoff of 1e-10 and an identity cutoff of 70%, and visualized using Easyfig v2.2.3.

*RNA sequencing and data analysis*

On the day 16, 10 mL of 0 and 6 g NaCl L^-1^ cultures were fixed with 10 % of ethanol containing 5 % of phenol and filtered on 0.22 µm polycarbonate filters (GE Water and Process Technologies). Filters were shock-frozen in liquid nitrogen and stored at -80 °C. Total RNA was isolated using RNeasy mini kit (Qiagen) and genomic DNA was depleted by TURBO DNA-*free*™ kit (Life Technologies). RNA Clean & Concentrator™ kit (Zymo Research) was used to purify and concentrate total RNA. Degradation of genomic DNA was ensured by quantitative real-time PCR using universal microbial 16S rRNA primers, RNA as a template and genomic cyanobacteral DNA as a positive control. Quality of RNA was verified by Bioanalyzer (Agilent Technologies) using total RNA 6000 Nano kit (Agilent Technologies). Ribosomal RNA removal (MICROBExpress™ Bacterial mRNA enrichment kit, Life Technologies), cDNA library preparation (Bacterial ScrptSeq Complete Kit, Illumina) and paird-end Illumina HiSeq sequencing was carried out at Institute for Molecular Medicine Finland (FIMM).

The quality of raw reads was checked using FastQC v0.11.4 (Andrews, 2010), and reads were demultiplexed with Trimmomatic v0.33 (Bolger et al., 2014). Clean reads were aligned to reference genomes using BWA-MEM v0.7.7 (Li, 2013) in paired-end mode with default parameters. SAMtools v1.2 (Li et al., 2009) was applied to convert the resulting SAM format to BAM format and filtered with BAM filter (Galaxy Version 0.5.7.1) to remove reads which were unmapped, less than 20 nt long, flagged as secondary alignments, marked as PCR duplicates or with low quality. The filtered BAM files were sorted by chromosomal coordinates with SAMtools v1.19 (Li et al., 2009) and converted to Wiggle format using the script bam2wig.py from the RseQC v2.4 package (Wang et al., 2012) in paired-end mode, and normalized to 1000000000 wigsum. The Wiggle format was then converted to Artemis (Carver et al., 2012) compatible genome coverage graphs using custom scripts available at https://github.com/housw/GRPutils. FeatureCounts v1.4.6.p5 (Liao et al., 2014) was used to quantify unambiguously aligned fragments to genomic features. Only paired-end reads which constituted a fragment size within 50 ~ 800 nt long was considered and chimeric fragments were excluded. The Galaxy instance of Freiburg University was used for read quality control and alignment (Afgan et al., 2016; Cock et al., 2013).

To exclude weakly expressed features, the counts were converted to Count Per Million (CPM), only features have a higher CPM (sum of CPM across all samples should be more than 3 and at least 3 samples had a CPM more than 1) were taken for differential expression analysis. Features belonging to rRNA and tRNA were excluded in this study. The differentially expressed genes between control (6 g L^-1^ NaCl) and treatment (0 g L^-1^ NaCl), were called using both edgeR (Robinson et al., 2010) and DESeq (Anders and Huber, 2010) following the simple design protocol (Anders et al., 2013). GO enrichment test was performed for differentially expressed genes using GOstats v2.40.0 (Falcon and Gentleman, 2007) with all the expressed genes as background. Semantic similarities of enriched GO terms were calculated using REVIGO online server (Supek et al., 2011).

**References**

Afgan, E., Baker, D., van den Beek, M., Blankenberg, D., Bouvier, D., Čech, M., et al. (2016). The Galaxy platform for accessible, reproducible and collaborative biomedical analyses: 2016 update. *Nucleic Acids Res* 44, W3–10. doi: 10.1093/nar/gkw343

Andrews, S. (2010). FastQC: A Quality Control tool for High Throughput Sequence Data. Available Online at http://www.bioinformatics.babraham.ac.uk/projects/fastqc/.

Anders, S., McCarthy, D. J., Chen, Y., Okoniewski, M., Smyth, G. K., Huber, W., and Robinson, M. D. (2013). Count-based differential expression analysis of RNA sequencing data using R and Bioconductor. *Nat Protocols* 8, 1765–1786.

Anders, S. and Huber, W. (2010). Differential expression analysis for sequence count data. Genome Biology 11, R106. doi: 10.1186/gb-2010-11-10-r106

Bolger, A. M., Lohse, M., and Usadel, B. (2014). Trimmomatic: a flexible trimmer for Illumina sequence data. *Bioinformatics* 30, 2114–2120.

Camacho, C., Coulouris, G., Avagyan, V. M. N., Papadopoulos, J., Bealer, K., and Madden, T. L. (2009). BLAST+: architecture and applications. *BMC Bioinformatics* 10, 421. doi: 10.1186/1471-2105-10-421

Carver, T., Harris, S. R., Berriman, M., Parkhill, J., and McQuillan, J. A. (2012). Artemis: an integrated platform for visualization and analysis of high-throughput sequence-based experimental data. *Bioinformatics* 28, 464–469.

Cock, P. J. A., Grüning, B. A., Paszkiewicz, K., and Pritchard, L. (2013). Galaxy tools and workflows for sequence analysis with applications in molecular plant pathology. *PeerJ* 1, e167. doi: 10.7717/peerj.167

Darling, A. C. E., Mau, B., Blattner, F. R., and Perna, N. T. (2004). Mauve: Multiple alignment of conserved genomic sequence with rearrangements. *Genome Res* 14, 1394–1403.

Darling, A. E., Mau, B., Perna, N. T. (2010)Perna progressiveMauve: Multiple genome alignment with gene gain, loss and rearrangement. *PLoS ONE* 5, e11147.

Eddy, S. R. (1998). Profile hidden Markov models. *Bioinformatics Review* 14, 755–763.

Edgar, R. C. (2004). MUSCLE: Multiple sequence alignment with high accuracy and high throughput. *Nucleic Acids Res* 32, 1792–1797.

Falcon, S., and Gentleman, R. (2007). Using GOstats to test gene lists for GO term association. *Bioinformatics* 23, 257–8.

Guy, L., Kultima, J. R., and Andersson, S. G. E. (2010). GenoPlotR: comparative gene and genome visualization in R. *Bioinformatics* 26, 2334–2335.

Kanehisa, M., Sato, Y., and Morishima, K. (2016). BlastKOALA and GhostKOALA: KEGG tools for functional characterization of genome and metagenome sequences. *J Mol Biol* 428, 726–731.

Lanfear, R., Calcott, B., Ho, S. Y. W., and Guindon, S. (2012). PartitionFinder: Combined selection of partitioning schemes and substitution models for phylogenetic analyses. Mol Biol Evol 29, 1695–1701.

Li, L., Stoeckert, C. J., and Roos, D. S. (2003). OrthoMCL: Identification of ortholog groups for eukaryotic genomes. *Genome Res* 13, 2178–2189.

Li, H. (2013). Aligning sequence reads, clone sequences and assembly contigs with BWA-MEM. Retrieved from http://arxiv.org/abs/1303.3997

Li, H., Handsaker, B., Wysoker, A., Fennell, T., Ruan, J., Homer, N., et al. (2009). The Sequence alignment/map format and SAMtools. *Bioinformatics* 25, 2078–9.

Liao, Y., Smyth, G. K., and Shi, W. (2014). FeatureCounts: An efficient general purpose program for assigning sequence reads to genomic features. *Bioinformatics* 30, 923–930.

Marchler-Bauer, A., Derbyshire, M. K., Gonzales, N. R., Lu, S., Chitsaz, F., Geer, L.Y., et al. (2015). CDD: NCBI’s conserved domain database. *Nucleic Acids Res* 43, 222–226.

Nawrocki, E. P., Kolbe, D. L., and Eddy, S.R. (2009). Infernal 1.0: inference of RNA alignments. *Bioinformatics* 25, 1335–1337.

Richter, M., Rosselló-Móra, R. (2009). Shifting the genomic gold standard for the prokaryotic species definition. *Proc Natl Acad Sci U S A* 10, 19126–19131.

Robinson, M. D., McCarthy, D. J., and Smyth, G. K. (2010). edgeR: a Bioconductor package for differential expression analysis of digital gene expression data. *Bioinformatics* 26, 139–140.

Stamatakis, A. (2014). RAxML version 8: A tool for phylogenetic analysis and post-analysis of large phylogenies. *Bioinformatics* 30, 1312–1313.

Supek, F., Bošnjak, M., Škunca, N., and Šmuc, T. (2011). REVIGO summarizes and visualizes long lists of Gene Ontology terms. *PLoS ONE* 6, e21800. doi: 10.1371/journal.pone.0021800

Varani, A. M., Siguier, P., Gourbeyre, E., Charneau, V., and Chandler, M. (2011). ISsaga is an ensemble of web-based methods for high throughput identification and semiautomatic annotation of insertion sequences in prokaryotic genomes. *Genome Biology* 12, R30. doi: 10.1186/gb-2011-12-3-r30

Wang, Y., Colemann-Derr, D., Chen, G., and Gu, Y. Q. (2015). OrthoVenn: a web server for genome wide comparison and annotation of orthologous clusters across multiple species. *Nucleic Acids Res* 43, W78–84. doi: 10.1093/nar/gkv487

Wang, L., Wang, S., and Li, W. (2012). RSeQC: quality control of RNA-seq experiments. *Bioinformatics* 28, 2184–5.

Wu, M., and Eisen, J. A. (2008). A simple, fast, and accurate method of phylogenomic inference. *Genome Biology* 9, R151. doi: 10.1186/gb-2008-9-10-r151
