# Supplementary material for: Comparative Genomics of the Baltic Sea Toxic Cyanobacteria Nodularia spumigena UHCC 0039 and Its Response to Varying Salinity
Source: Front Microbiol. 2018 Mar 8;9:356. doi: 10.3389/fmicb.2018.00356 (PMC5853447; doi:10.3389/fmicb.2018.00356)
Supplement: Supplementary file 4 [file Image4.PDF]

(a) (b)

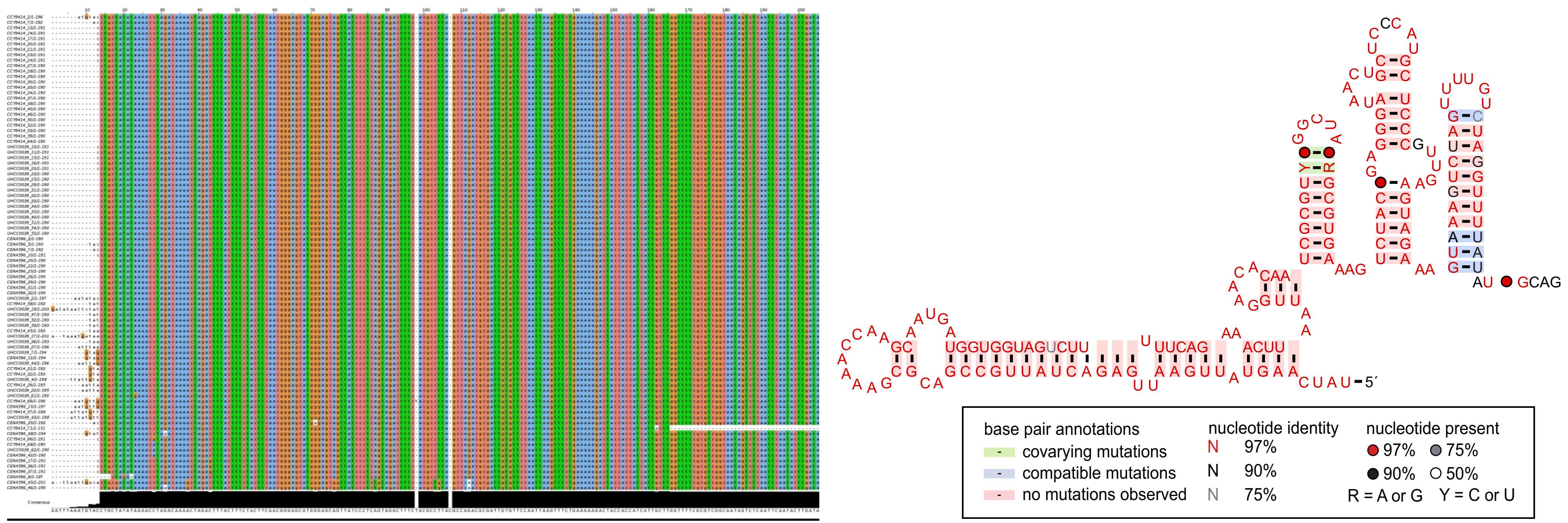

(c)

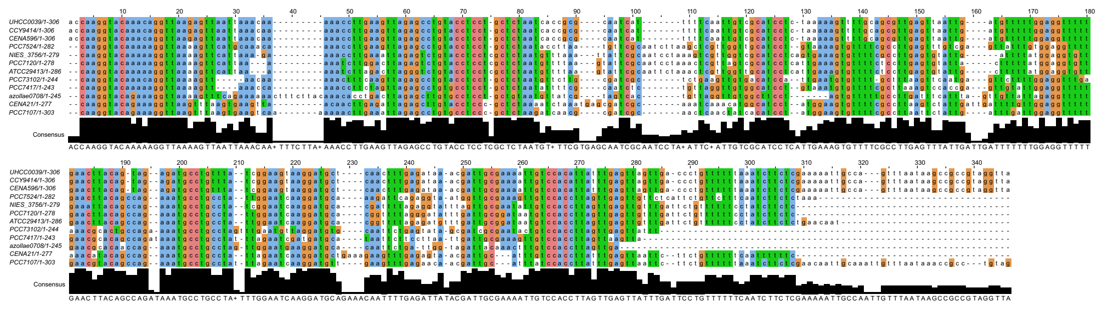

(d)

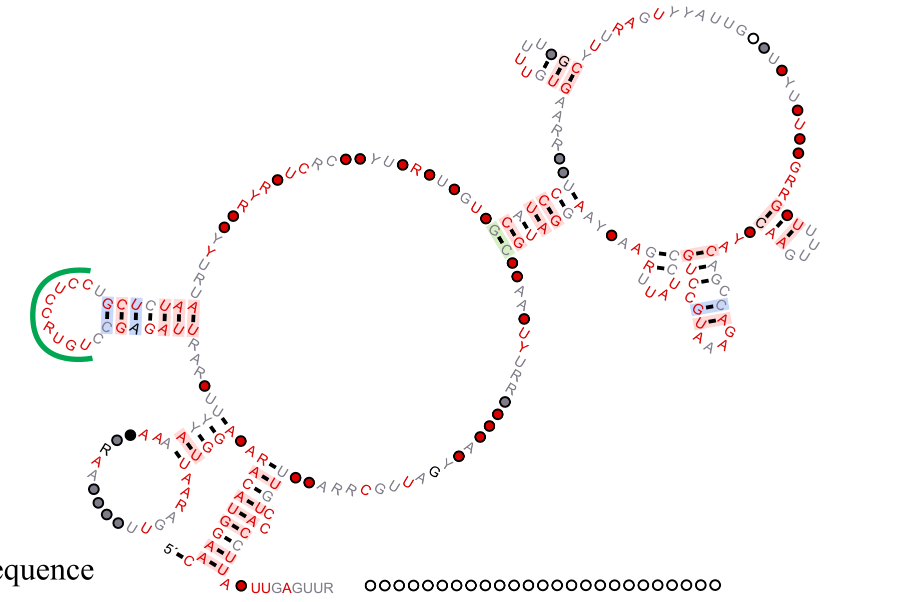

Figure S4 Selected expressed non-coding RNAs and the conserved secondary structures. Sequence alignment and secondary structure of DGR1 (a, b) and a conserved ncRNA nss009 (c, d).
